# Keratin-based protection of enamel against acid-erosion

**DOI:** 10.3389/fdmed.2026.1869803

**Published:** 2026-07-13

**Authors:** Simran Mistry, Prema Sukumaran, Sherif Elsharkawy

**Affiliations:** Centre for Clinical, Translational and Oral Sciences, Faculty of Dentistry, Oral and Clinical Sciences, King’s College London, London, United Kingdom

**Keywords:** dental enamel, erosion, fluoride, keratin, microhardness, surface profilometry

## Abstract

Enamel erosion is a prevalent and growing oral health concern, affecting approximately 20%–45% of the adult population, with incidence expected to increase. Current preventative strategies rely predominantly on fluoride-based interventions, which, while effective, are not without limitations, particularly in the context of erosive challenges. Consequently, there is an increasing interest in alternative or adjunctive approaches for protecting enamel against acid-induced damage. This study investigated the protective potential of a keratin-based surface treatment against acid-induced enamel erosion and compared its performance with sodium fluoride at clinically relevant concentrations. Enamel specimens from extracted human molars were treated with keratin, sodium fluoride (1,450, 5,000, or 22,600 ppm), artificial saliva, or deionised water, and subsequently exposed to citric or hydrochloric acid under *in vitro* conditions. Enamel surface microhardness, surface profilometry, and scanning electron microscopy were used to assess hardness retention, surface loss, and morphological changes. Following acid exposure, keratin-treated specimens exhibited greater hardness retention than control groups and lower fluoride concentrations under both erosion models, with performance approaching that observed in the 22,600 ppm fluoride group. Profilometric analysis indicated reduced surface loss in treated groups relative to controls, although differences were not statistically significant. SEM observations supported these findings, demonstrating improved preservation of surface and subsurface enamel structure in keratin- and high-fluoride-treated samples. Within the limitations of this *in vitro* study, keratin demonstrated consistent protective effects against enamel erosion, supporting further investigation as a potential biomaterial for erosion prevention under clinically relevant conditions.

## 1 Introduction

Dental enamel is the most highly mineralised and mechanically resilient tissue in the human body. Its hierarchical arrangement of densely packed hydroxyapatite crystallites organised into prism-based units confers exceptional hardness, wear resistance and fracture tolerance (1, 2). At the tooth surface, this architecture enables enamel to act as the primary protective barrier for the underlying dentine-pulp complex against mechanical, thermal and chemical challenges (3).

Despite this protective role, enamel is inherently susceptible to chemical degradation owing to its high mineral content (4). Exposure to extrinsic (e.g., dietary acids) or intrinsic acids (e.g., gastric reflux or regurgitation) initiates demineralisation of hydroxyapatite crystals, leading to surface softening and increased porosity. Repeated acidic episodes progressively weaken the softened layer, leaving enamel increasingly vulnerable to abrasive and attritional forces (5). The combined effect of acid-mediated softening followed by mechanical removal results in erosive tooth wear, the progressive and irreversible loss of enamel in the absence of bacterial involvement (6). As enamel is acellular and incapable of remodelling once formed, mineral loss is permanent (7); cumulative erosion over time alters tooth morphology, increases hypersensitivity and negatively affects aesthetics and oral function. With global prevalence estimates indicating that 20%–45% of adults exhibit signs of erosive tooth wear, it represents a significant and growing oral health concern (8).

Current interventions for erosive tooth wear are largely preventative, focusing on limiting enamel mineral loss during acid exposure rather than restoring lost enamel. Fluoride remains the gold standard agent and is primarily delivered through toothpaste, gel and varnish formulations (9). Fluoride concentrations vary considerably between these products, with conventional over-the-counter toothpastes typically containing 1,000–1,500 ppm fluoride, prescription strength formulations containing approximately 5,000 ppm fluoride and professionally applied fluoride varnishes approximately 22,600 ppm fluoride (10). Its protective effect arises from the fluoridation of hydroxyapatite and the deposition of calcium fluoride-like precipitates on the enamel surface, which enhance short-term resistance to demineralisation (11). The protective CaF_2_ -like layer formed on the enamel surface is relatively superficial and may be progressively reduced by repeated acidic and abrasive challenges, potentially limiting the durability of protection under highly erosive challenges (12). Although fluoride-based therapies remain highly effective and represent the cornerstone of erosion prevention, growing interest has emerged in biomaterial-based strategies capable of providing complementary or alternative, more biomimetically aligned mechanisms of enamel protection during erosive challenge, particularly under conditions where conventional fluoride-based protection may be reduced. Interest in non-fluoride approaches has also increased in response to concerns regarding excessive fluoride exposure associated with prolonged use of high-concentration fluoride formulations.

Limitations of conventional fluoride treatments have highlighted the need for novel strategies to protect enamel against acid-induced erosion in erosive tooth wear. In response, a range of biomaterial-based alternatives and adjunct therapies have been explored, with several materials demonstrating potential to form protective surface coatings, buffer acidic environments and support mineral deposition (13, 14). These include nano- and micro-sized hydroxyapatite particles, casein phosphopeptide-amorphous calcium phosphate (CPP-ACP), bioactive glass and chitosan (15, 41). Nano-hydroxyapatite has attracted particular interest due to its chemical and structural similarity to biological apatite. Previous *in vitro* and *in situ* studies have demonstrated that nano-hydroxyapatite containing formulations can reduce enamel softening, decrease erosion depth and preserve enamel surface morphology under acidic conditions, potentially through deposition of mineral-like particles and increased calcium and phosphate ion availability at the enamel surface (16, 17). However, findings remain inconsistent under strong demineralizing conditions and concerns remain regarding the durability of the deposited mineral layer during repeat acid exposure (18).

CPP-ACP stabilises calcium and phosphate ions to create a supersaturated mineral reservoir on the enamel surface, though its effectiveness is inconsistent across studies and its milk-derived origin and cost restricts wider use (19). Bioactive glass releases calcium, phosphate and silicon ions that promote formation of an enamel-like mineral layer; however, this layer develops slowly and may be disrupted under typical oral conditions (20). Chitosan can form an adsorbed protective film and enhance fluoride retention, but challenges related to production cost, sourcing and limited *in vivo* evidence restrict its clinical translation (21). While these biomaterials represent important advances in erosive tooth wear research, their clinical translation remains limited. Collectively, these limitations highlight the need for alternative, biocompatible and practical biomaterials capable of improving enamel protection during erosive episodes.

Keratin, a naturally occurring structural protein abundant in wool, hair and feathers, has emerged as a promising candidate for dental biomaterial development (22, 23). Its high cysteine content enables disulphide cross-linking which confers notable mechanical strength and chemical stability, including resistance to weak acids (24). These properties, together with its biocompatibility and low immunogenicity, have supported its use in a wide range of biomedical applications including wound repair, haemostasis, drug delivery and tissue engineering (23, 25, 37). Keratin also demonstrates relevance to dental biology, with hair-derived keratins identified within the organic matrix of mature enamel and keratin gene mutations associated with enamel defects (26).

Despite its favourable characteristics, keratin remains largely unexplored as a topical agent for protecting enamel against acid-induced erosion. Given its stability, hydrophobicity and capacity to form adherent surface layers, keratin may offer a novel approach to enhancing enamel protection under erosive conditions. This study therefore investigates keratin as a potential enamel-protective biomaterial and evaluates its performance against intrinsic and extrinsic acid challenges, in comparison with conventional fluoride treatment. Keratin was assessed as a stand-alone topical coating agent rather than as a fluoride carrier or combination system. The null hypothesis was that keratin application would not produce significant differences in enamel surface hardness or surface loss compared with conventional fluoride treatment.

## 2 Materials and methods

### 2.1 Enamel sample preparation

Forty caries-free extracted human molars were collected under ethical approval (REC:12/LO/1836), disinfected in 5% sodium hypochlorite for 3 days (27) and stored in deionised water prior to use. The roots were removed, and the crowns were then sectioned mesiodistally using a precision saw (Buehler® Isomet®1000, Illinois, USA) yielding 80 enamel sections (40 buccal, 40 palatal/lingual). Samples were ultrasonicated for 15 min to remove debris and mounted in self-curing polymethyl methacrylate (PMMA) (Oracryl, Bracon, East Sussex, UK) using a customised silicone mould (25 mm diameter × 20 mm height) (28). When mounting, the buccal or palatal/lingual surfaces were oriented downwards.

After 24 h of curing, mounted samples were sequentially polished using silicon carbide papers (P500–4,000 grits; VersoCit, Struers, Denmark) on a water-cooled polishing machine (Struers, Denmark) operating at 150 rpm under a 10 N load (28, 29). This achieved a flat enamel surface (surface flatness ± 0.2 µm) suitable for indentation and profilometry. Polished samples were ultrasonicated for 15 min, air-dried, then examined under a digital microscope (Keyence Corporation, Osaka, Japan) at ×300 magnification. Samples with visible defects, including white spot lesions or cracks, were excluded and replaced.

**Figure 1:**
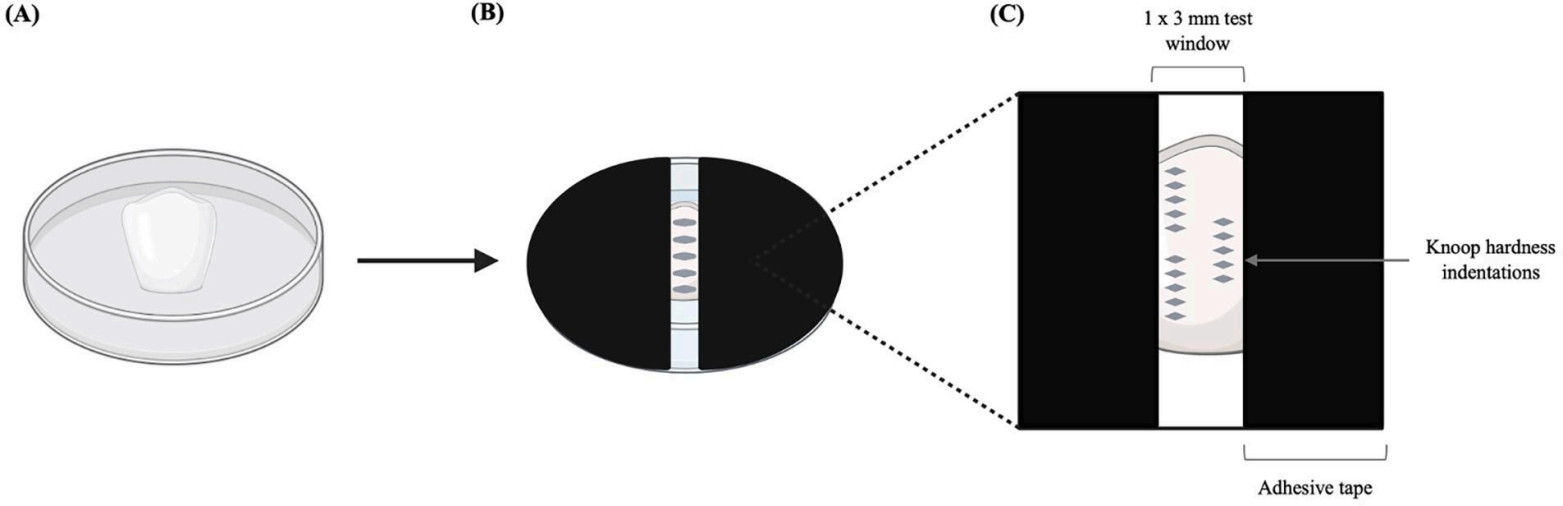
Schematic representation of the enamel test window used for surface analyses. **(A)** Enamel sections were embedded in PMMA acrylic resin and polished to produce a flat enamel surface. **(B)** Adhesive tape was applied to create a central 1 × 3 mm exposed enamel test window. **(C)** Enlarged view of the test window showing the analysis region. The exposed enamel area was used for Knoop microhardness measurements, profilometric step-height analysis and SEM evaluation. Five Knoop hardness indentations were placed within the exposed enamel region at each assessment time point (baseline, post-treatment and post-acid exposure), with indentations spaced 100 gm apart and positioned in different regions of the test window to avoid overlap with previous measurements. Created with BioRender.com.

Adhesive tape was applied to define a 1.0 × 3.0 mm central test window with reference regions on either side (Figure 1). Baseline microhardness was measured using a Knoop microhardness tester (Duramin-5, Struers Ltd.). Five indentations (0.1 kgf, 10 s dwell) were placed 100 µm apart within the test window and the long diagonals were measured microscopically (×300). The mean Knoop Hardness Number (KHN) was calculated from the five measurements. Eight samples outside the predefined baseline range of 270–400 KHN were excluded. An additional 12 enamel samples were reserved for SEM imaging and excluded from all quantitative analyses.

### 2.2 Experimental design and sample allocation

Following baseline characterisation, 60 eligible enamel sections were treated as independent experimental units, irrespective of their tooth of origin. Each buccal and palatal/lingual half was assigned a unique identifier and randomised individually to one of six experimental treatment groups using the RAND function in Microsoft Excel (*n* = 10 per group).

The study employed a two-stage experimental structure. First, samples were allocated to one of six treatment conditions (Keratin 10%, Sodium Fluoride at 1,450, 5,000 or 22,600 ppm, artificial saliva or deionised water). Second, each treatment group was subsequently divided for exposure to either citric acid (extrinsic erosion model) or hydrochloric acid (intrinsic erosion model), resulting in *n* = 5 samples per acid condition within each treatment group.

Because allocation was performed at the level of the individual enamel section, halves originating from the same donor tooth could, by chance, be assigned to the same or different treatment group and no paired or clustered structure was assumed in the statistical analysis.

### 2.3 Sample size calculation

Sample size estimation was performed using G*Power version 3.1 using data from a preliminary study, which indicated that 4 samples per group would provide 80% power to detect differences in enamel microhardness and step height. The final sample size used in this study (*N* = 60, *n* = 10 per treatment group; *n* = 5 per acid condition per group) exceeded this minimum required and was therefore considered sufficient to test the study hypothesis.

### 2.4 Keratin extraction from sheep wool

Ten grams of sheep wool (Hardicott Shetland Fleece, Leigh Barton Farm, Devon) were cleaned several times using deionised water, then air-dried. Lipids and waxes were removed by Soxhlet extraction (hexane:dichloromethane 1:1 v/v, 6 h at 65°C) (42). The wool was then treated in a solution containing 180 mL of 7M urea (Urea powder, ≥98%, Sigma Aldrich, Dorset, UK), 6 g of sodium dodecyl sulphate (Sigma Aldrich, Dorset, UK) and 15 mL of 2-mercaptoethanol (2-Mercaptoethanol, ≥99%, Sigma Aldrich, Dorset, UK) at 50°C for 24 h with the mixture maintained at pH 7.0 (28, 30). The suspension was filtered, centrifuged (6,000 rpm, 30 min, 4°C) and the supernatant dialysed (5 kDa MWCO) against deionised water for 3 days, with twice-daily water changes. The purified keratin was freeze-dried to yield a white fibrous powder (28, 31).

### 2.5 Preparation of artificial saliva

Artificial saliva was prepared by dissolving the following components in 1,000 mL of ultrapure water under continuous stirring: 0.5146 g CaCl₂·2H₂O, 0.0952 g MgCl₂, 2.722 g KH₂PO₄, 11.826 g KCl, and 26.03 g HEPES (all Sigma Aldrich, Dorset, UK). The pH was adjusted to 7.0 with 0.1 M NaOH (Sigma Aldrich, Dorset, UK) and then stored at 4°C (32).

### 2.6 Application of keratin and NaF solutions

Samples were allocated to six treatment groups (*n* = 10 per group): keratin, sodium fluoride at 1,450, 5,000 or 22,600 ppm, artificial saliva and deionised water. Samples within each treatment were divided equally and exposed to either citric acid (extrinsic erosion model) or hydrochloric acid (intrinsic erosion model) (*n* = 5 per acid condition).

For the keratin group, a 10% (w/v) aqueous keratin solution was prepared by dissolving 10 mg lyophilised keratin in 100 µL ultrapure water (pH 7.0). The resulting formulation formed an aqueous solution suitable for topical application onto the enamel surface. A single, uniform coat (20 µL) was applied to each enamel window using a dental applicator brush and allowed to dry in an incubator at 34.0°C for 3 min (28).

The selection of both the 10% keratin concentration was informed by previous experimental work within our research group, in which varying concentrations (3%, 5%, 10% and 20%) were systematically evaluated (33). Based on these findings, the 10% formulation provided the most favourable balance between material stability, ease of handling and mineralisation potential.

The application volume was determined through a separate preliminary optimisation study in which 10, 20 and 30 µL volumes of keratin solution were evaluated based on their effects on surface step height. The results demonstrated that 10 µL was insufficient to achieve complete coverage of the exposed enamel area, whereas no appreciable difference in step height was observed between the 20 and 30 µL groups. As 30 µL required a longer drying time and resulted in greater material accumulation on the enamel surface, 20 µL was selected as it provided complete surface coverage while maintaining favourable handling characteristics (Supplementary Figure S1).

Sodium fluoride (NaF; Sigma Aldrich, Dorset, UK) solutions containing 1,450, 5,000 or 22,600 ppm NaF were freshly prepared in deionised water and applied using the same application and drying protocol. Following treatment application and drying, all treated samples were transferred into individual beakers containing 50 mL artificial saliva. Control samples did not receive any topical treatment and were instead placed into individual beakers containing 50 mL of either deionised water or artificial saliva. All beakers were incubated at 37°C with agitation at 60 rpm for 24 h to simulate intraoral conditions.

After incubation, samples were rinsed with deionised water for 15 s, ultrasonicated for 2 min at 40 kHz, air-dried overnight, and the adhesive tape was removed.

### 2.7 Surface profilometry and microhardness

Non-contact white light confocal profilometry (XYRIS 2000, TaiCaan, UK) was used to scan each enamel surface (5 × 5 mm area, 10 µm intervals). Mean step height relative to adjacent untreated enamel was quantified using metrological analysis software (Mountains®9, MountainsMap®, Digital Surf, France) (27). Step height of eroded lesions was calculated in accordance with ISO 5436-1 standards, using the two reference zones located on either side of the test window.

Post-treatment enamel surface microhardness was assessed using the same Knoop parameters as those used for baseline measurements. Following microhardness assessment, adhesive tape was reapplied to redefine the test window prior to acid exposure.

### 2.8 Preparation of acid

Citric acid (extrinsic erosion model): 3 g anhydrous citric acid (Sigma Aldrich, Dorset, UK) was dissolved in 800 mL deionised water. The pH was adjusted to 2.7 with 0.1 M NaOH (Sigma Aldrich, Dorset, UK), and the volume made up to 1L.

Hydrochloric acid (intrinsic erosion model): 0.01 M HCl (Sigma Aldrich, Dorset, UK) was prepared by diluting 10 mL 1 M HCl stock to 1L using deionised water.

### 2.9 Enamel erosion simulation

The citric acid and hydrochloric exposure protocols were selected to represent *in vitro* models of extrinsic and intrinsic erosion, respectively. Repeated citric acid cycles were used to simulate the prolonged reduction in oral pH that may occur following consumption of acidic food and beverages. In contrast, the hydrochloric acid model consisted of a single, shorter exposure to reflect the transient contact of gastric acid with enamel during intrinsic erosive events. These protocols were based on previously published erosion models (27).

For citric acid exposure, samples were individually immersed in 20 mL acid at room temperature and agitated at 62 rpm for four 5 min cycles using an orbital shaker (Stuart Mini Orbital Shaker SSM1, Bibby Scientific, England). Between each cycle, samples were rinsed with deionised water, and the acid solution was replaced (28).

Hydrochloric acid exposure consisted of a single 2 min cycle under identical agitation. Following acid exposure, all samples were rinsed, ultrasonicated for 2 min, and air-dried for 24 h. The ultrasonication duration was selected based on preliminary pilot work comparing 2 and 10 min of ultrasonication. As no significant differences in step height were observed between the two time points, the shorter 2 min duration was selected and applied consistently throughout the study.

### 2.10 Post-acid surface profilometry and microhardness

Step height was remeasured by profilometry under identical conditions to the post-treatment application stage. Final surface microhardness was determined using the same parameters as baseline testing and values were expressed as a percentage of baseline KHN.

### 2.11 Scanning electron microscopy (SEM)

Two samples per acid condition were randomly selected from each treatment group for SEM analysis (one for surface imaging and one for cross-sectional analysis). After gold sputter-coating, samples were imaged using a field-emission scanning electron microscope (JSM-7800F Prime, JEOL, Japan) at 10 kV and magnifications ranging from ×750 to ×10,000 to assess enamel surface and subsurface morphology. SEM was used for qualitative assessment only and was not used in quantitative statistical analysis.

### 2.12 Statistical analysis

Microhardness and profilometry data were analysed using GraphPad Prism (version 9.5.0; GraphPad Software, San Diego, USA). Microhardness values were normalised to baseline and expressed as a percentage retention (mean ± standard deviation). Statistical analyses were performed separately for the citric acid and hydrochloric acid erosion models. Normality was evaluated with the Shapiro–Wilk test. Normally distributed data were compared using one-way ANOVA with Dunnett's *post hoc* test, while non-normally distributed data were analysed using the Kruskal–Wallis test with Dunn's multiple comparisons. Statistical significance was set at *p* < 0.05.

## 3 Results

### 3.1 Enamel surface microhardness

**Figure 2:**
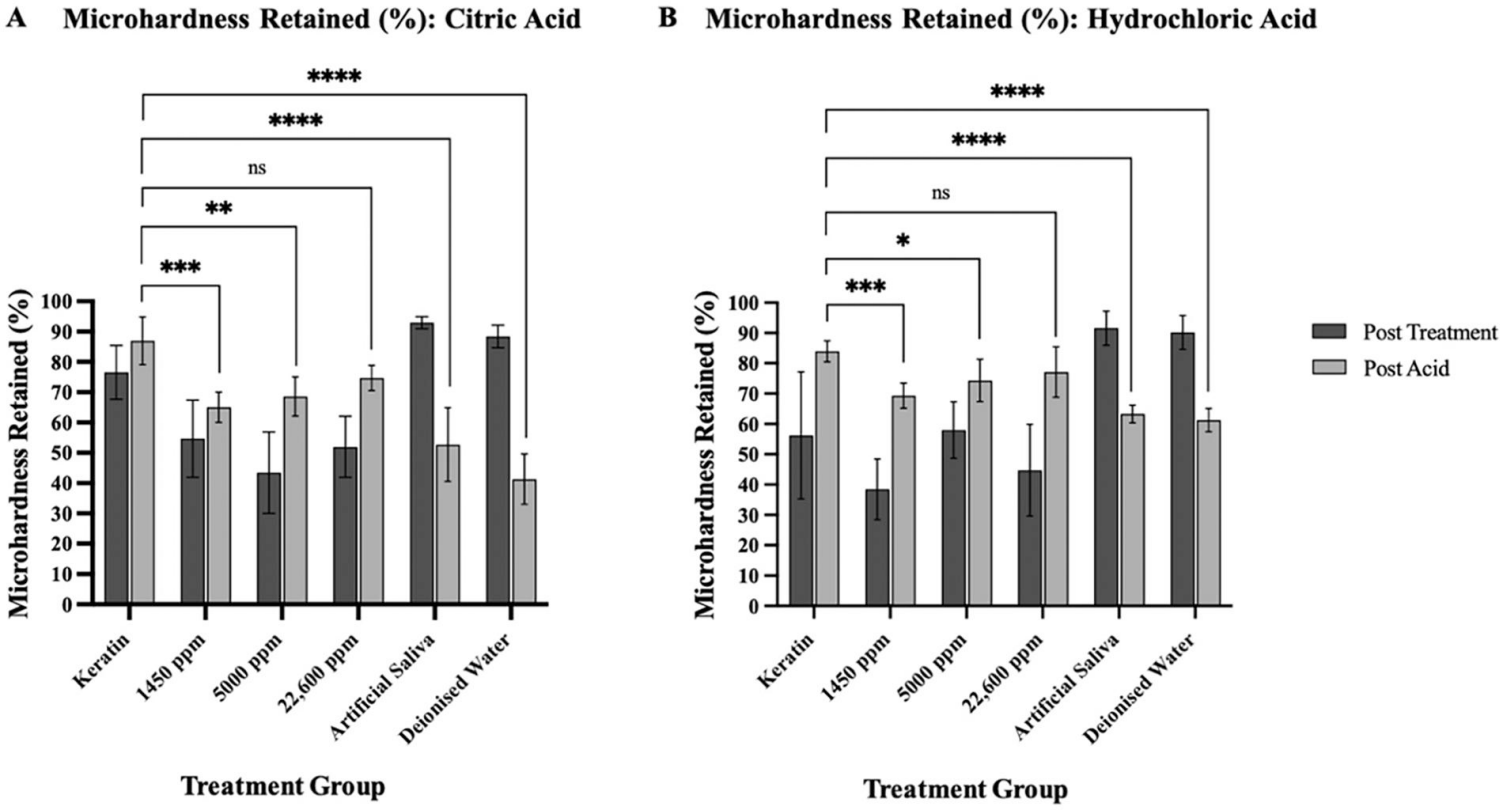
Percentage of baseline enamel hardness retained following treatment application and subsequent acid exposure. Surface microhardness was measured within the defined enamel treatment window using a Knoop indenter (0.1 kgf load and 10 s dwell time). Values were normalised to each sample's baseline hardness and expressed as a percentage. Bars represent mean normalised microhardness values (±SD) post treatment and post acid exposure for each group (*n* = 5). **(A)** Shows results following citric acid exposure; **(B)** Shows results following hydrochloric acid exposure. Statistical comparisons were made relative to the keratin-treated group using one-way ANOVA with Dunnett's *post hoc* test. *p < 0.05, **p < 0.01, ***p < 0.001, ****p < 0.0001; ns = not significant.

Enamel surface microhardness was measured at baseline, post-treatment application and post-acid exposure, with raw KHN values presented in Supplementary Table S3. Post-treatment and post-acid Knoop Hardness Numbers (KHN) were normalised to baseline values and expressed as percentage hardness retention (Figure 2; Supplementary Table S1).

All groups demonstrated a reduction in percentage hardness relative to baseline following treatment application, with greater reductions observed in treated groups than in controls.

Under citric acid challenge (Figure 2A), percentage hardness retention increased relative to post-treatment values in the keratin- and fluoride- treated groups, while further reductions were observed in the artificial saliva and deionised water control groups. The keratin-treated group retained 86.96% ± 7.91 of baseline hardness, which was significantly higher than the 1,450-ppm fluoride group (*p* ≤ 0.001), the 5,000 ppm fluoride group (*p* ≤ 0.01), and both control groups (*p* ≤ 0.0001), but was not significantly different from 22,600 ppm fluoride (ns).

Following hydrochloric acid exposure (Figure 2B), a similar pattern was observed, with increased percentage hardness retention relative to post-treatment values in the keratin- and fluoride-treated groups, whereas further decreases were observed in the control groups. The keratin-treated group retained 69.94% ± 6.48 of baseline hardness, which was significantly higher than the 1,450 ppm and 5,000 ppm fluoride groups (*p* ≤ 0.05), and both control groups (*p* ≤ 0.0001), but not significantly different from the 22,600 ppm fluoride group (ns).

### 3.2 Step height analysis

**Figure 3:**
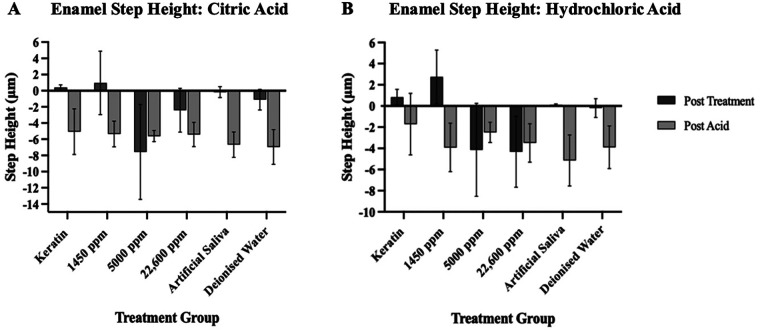
Enamel step height measurements following treatment application and acid exposure (citric acid in **A**; hydrochloric acid in **B**). Enamel surface profiles were obtained using non-contact white light profilometry and analysed in accordance with ISO 5436-1 standards. Step height was measured within the treated enamel window and compared to adjacent untreated regions. Bars represent mean step height values (±SD) recorded post treatment and post acid exposure for each group (*n* = 5). No statically significant differences were found, and significance markers have been omitted for clarity.

Surface profilometry was used to quantify step height changes within the enamel treatment window following treatment application and acid exposure (Figure 3; Supplementary Table S2). Positive step height values indicate surface elevation relative to untreated enamel, whereas negative values indicate surface loss.

For samples assigned to subsequent citric acid exposure, post treatment measurements showed positive mean step height values for the keratin-treated (0.41 μm ± 0.32) and 1,450 ppm fluoride groups (0.95 μm ± 3.90), while all other groups showed negative mean values (Figure 3A). Following acid exposure, all groups exhibited negative step height values (−5.07 to −6.96 µm), with keratin treated samples demonstrated the lowest mean surface loss (−5.07 μm ± 2.82). Control groups showed the greatest mean surface loss; however, no statistically significant differences were observed between groups (Kruskal–Wallis, *p* = 0.3997). Pairwise comparisons using Dunn's multiple-comparisons test also revealed no significant differences between the keratin-treated group and any other treatment group (adjusted *p* > 0.9999 for all comparisons).

Figure 3B shows corresponding data for samples assigned to subsequent hydrochloric acid exposure. Post-treatment measurements showed positive mean step height values for the keratin-treated, 1,450-ppm fluoride and artificial saliva groups, whereas the remaining groups exhibited negative mean values. Following hydrochloric acid exposure, all groups exhibited negative step height values. The keratin-treated group showed the lowest mean surface loss (−1.72 μm ± 2.90), whereas the artificial saliva group showed the greatest loss (−5.15 μm ± 2.41). No statistically significant differences were observed between groups [one-way ANOVA, F (5,24) = 1.558, *p* = 0.2097]. Pairwise comparisons using Dunnett's multiple -comparisons test likewise demonstrated no significant differences between the keratin-treated group and any other treatment group (adjusted *p* = 0.0727–0.9690).

### 3.3 Scanning electron microscopy (SEM)

**Figure 4:**
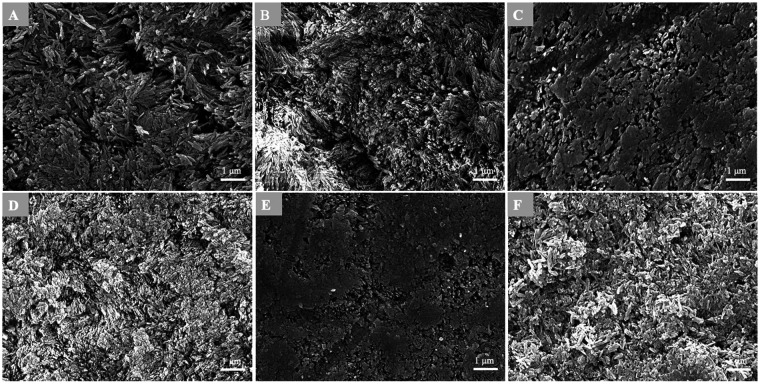
Scanning electron microscopy (SEM) images of treated enamel window following citric acid exposure. SEM images are shown for each treatment group: **(A)** deionised water, **(B)** artificial saliva, **(C)** 1,450 ppm fluoride, **(D)** 5,000 ppm fluoride, **(E)** 22,600 ppm fluoride and **(F)** keratin. Images were captured at ×10,000 magnification, with a working distance of 10 mm, acceleration voltage of 10.0 kV, and a probe current of 6.

**Figure 5:**
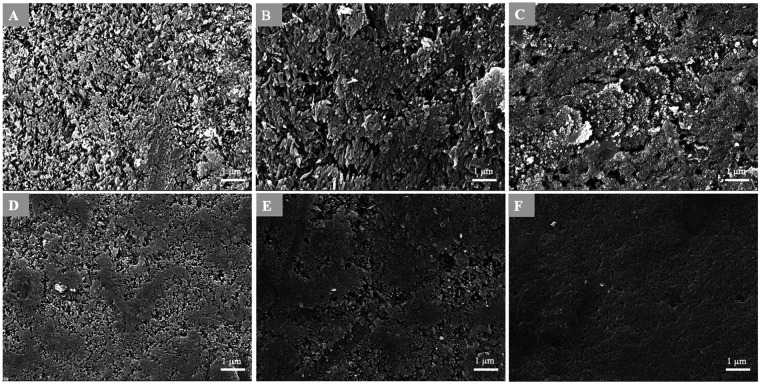
Scanning electron microscopy (SEM) images of treated enamel window following hydrochloric acid exposure. SEM images are shown for each treatment group: **(A)** deionised water, **(B)** artificial saliva, **(C)** 1,450 ppm fluoride, **(D)** 5,000 ppm fluoride, **(E)** 22,600 ppm fluoride and **(F)** keratin. Images were captured at ×10,000 magnification, with a working distance of 10 mm, acceleration voltage of 10.0 kV, and a probe current of 6.

SEM images of enamel surfaces following citric acid and hydrochloric acid exposure are shown in Figures 4, 5. As SEM was used for qualitative assessment only, one specimen from each treatment group was randomly selected for surface imaging and one specimen for cross-sectional imaging within both erosion models.

Following citric acid exposure (Figure 4), control samples (deionised water and artificial saliva) exhibited porous, irregular surfaces with visible enamel rods and numerous dark, structureless regions. Fluoride-treated samples appeared progressively smoother surfaces with increasing concentration. The 22,600 ppm fluoride group displayed a relatively uniform surface layer with minimal porosity. Keratin-treated enamel displayed a tightly packed surface with few porous regions and scattered light elevations on the enamel surface.

Following hydrochloric acid exposure (Figure 5), both control groups again showed rough, porous structures and exposed enamel rods. The fluoride-treated samples demonstrated improved surface integrity with increasing fluoride concentration, with the 22,600 ppm group exhibiting a continuous surface with minimal porosity. The keratin-treated sample displayed a smooth, uniform surface morphology with little evidence of exposed enamel rods.

**Figure 6:**
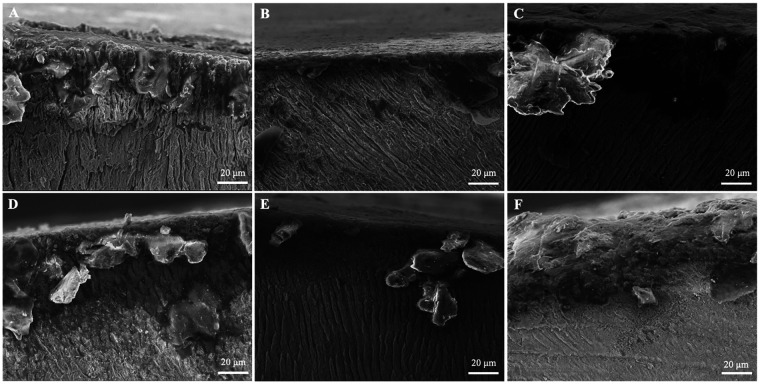
Scanning electron microscopy (SEM) images of treated enamel window following hydrochloric acid exposure. SEM images are shown for each treatment group: **(A)** deionised water, **(B)** artificial saliva, **(C)** 1,450 ppm fluoride, **(D)** 5,000 ppm fluoride, **(E)** 22,600 ppm fluoride and **(F)** keratin. Images were captured at ×750 magnification, with a working distance of 10 mm, acceleration voltage of 10.0 kV, and a probe current of 6.

Cross-sectional SEM images (Figures 5, 6) revealed structural differences within the enamel layers. In both erosion models, control samples displayed substantial surface and subsurface degradation, including disrupted or porous enamel rod structures. Fluoride treatments provided progressively greater preservation of enamel structure with increasing concentration, with the 22,600 ppm group showing a compact, well-defined surface layer and densely packed subsurface rods. Keratin-treated enamel exhibited an intact surface with a distinct superficial layer and uniform subsurface enamel, with shorter and more rounded rods. The observed superficial layer may represent retained treatment material and/or associated mineral deposits; however, its composition was not directly confirmed in the present study.

## 4 Discussion

This study evaluated the ability of a keratin-based surface layer to protect human enamel from erosion caused by intrinsic (hydrochloric acid) and extrinsic (citric acid) acids under simulated acid erosion cycles. Keratin's performance was compared with sodium fluoride treatments at three clinically relevant concentrations, alongside artificial saliva and deionised water controls. Enamel surface microhardness, profilometric step-height analysis, and scanning electron microscopy (SEM) collectively provided insight into keratin's protective potential and its performance relative to established fluoride-based approaches.

Enamel surface microhardness was a key measure in this study, as it is widely used to quantify enamel softening and thus assess the effectiveness of protective interventions (34). Measurements were taken at baseline, after treatment application and following acid exposure, allowing changes in surface properties to be monitored across each stage of the experimental cycle. To account for natural variability in the initial hardness of specimens and enable accurate comparison between groups, post-treatment and post-acid exposure values were normalised and expressed as the percentage of baseline hardness retained. Higher percentages (approaching 100%) indicate greater preservation of enamel integrity, whereas lower percentages reflect acid-induced softening associated with hydroxyapatite dissolution.

Comparisons between groups focused on the post-treatment and post-acid stages. Assessing these two timepoints enabled us to characterise the formation of treatment-derived surface layers and evaluate their resilience to subsequent acid challenge, allowing evaluation of both the immediate and protective effects of each intervention.

At the post-treatment stage, all groups exhibited reductions in surface microhardness relative to baseline, indicating apparent surface softening (Figure 1) (28). The greatest reductions were observed in the treatment groups, which retained an average of 48.5% of baseline hardness, compared with 83.2% in the control groups. Although the applied treatments were intended to enhance enamel resistance, such reductions at this stage are consistent with previous reports and do not necessarily reflect true demineralisation of the underlying enamel (28). The most plausible explanation for the reduced microhardness at this stage is the formation of relatively soft, treatment-derived surface layers rather than mineral loss from the enamel substrate (40). As these layers were incubated for only 24 h, they were unlikely to be fully mineralised and would therefore be expected to possess mechanical properties distinct from those of mature enamel. The reduced Knoop hardness values observed at this stage may reflect the properties of the newly formed surface layer, at least in part, rather than the underlying enamel alone.

Profilometric analysis demonstrated that these treatment layers were up to 5 µm thick (Figure 3), whereas the Knoop microhardness indenter is known to penetrate only a few microns under standard testing loads (35). Consequently, post-treatment measurements likely reflected indentation within these superficial layers, which are softer than enamel, rather than the underlying enamel itself (39). In contrast, control specimens exhibited substantially thinner surface films, allowing more direct contact between the indenter and the enamel. This accounts for the higher retained microhardness observed in control samples at the post-treatment stage. SEM imaging supported this interpretation, revealing distinct surface coatings on treated enamel that were absent or minimal in control samples (Figures 4, 5).

Following acid exposure to both citric (extrinsic) and hydrochloric (intrinsic) acids, surface microhardness increased in the fluoride- and keratin-treated groups relative to post-treatment values, whereas control specimens exhibited further softening. This indicates greater stability of the treatment-modified enamel surface relative to untreated controls during erosive challenge, while the continued loss in controls confirms the validity of the erosion model.

The observed increase in microhardness following acid exposure in the treated groups is best explained by partial removal or thinning of the initially softened surface layer, allowing the Knoop indenter to engage a harder underlying surface. In fluoride-treated samples, this likely reflects dissolution of calcium-fluoride-like deposits and increased exposure of a more acid-resistant fluorapatite surface (36). In keratin-treated samples, the proteinaceous surface layer was similarly disrupted during acid exposure. This interpretation is supported by SEM imaging, which demonstrated disruption of the post-treatment surface layers and the development of erosive surface features following acid exposure.

Among the fluoride-treated groups, a clear dose-dependent relationship was observed, with higher fluoride concentrations producing progressively greater microhardness retention. This trend is consistent with increased formation and surface coverage of calcium-fluoride–like deposits at higher concentrations, enhancing fluoride availability at the enamel surface during acid exposure and improving resistance to demineralisation (36).

Keratin-treated samples showed the highest mean microhardness retention under both the extrinsic (86%) and intrinsic (69%) erosion models, with values significantly higher than those of the control groups and all lower-concentration fluoride treatments. No significant difference was detected between keratin and the 22,600 ppm fluoride group in either model. As 22,600 ppm fluoride is representative of professionally applied fluoride varnish formulations, this finding is of particular clinical relevance. Keratin also outperformed the 1,450 ppm and 5,000 ppm fluoride groups, which are representative of conventional over the counter and prescription strength fluoride products, respectively. These findings suggest that keratin may offer a level of protection approaching that of professionally applied high-dose fluoride varnish while exceeding that achieved by lower concentration fluoride formulations routinely used by patients. If these effects can be replicated *in situ* and clinically, keratin-based formulations may offer a promising and potentially more accessible approach to erosion prevention. However, further research is required to determine the feasibility, durability and effectiveness of such applications within the oral environment.

A key strength of the study was the inclusion of both citric acid and hydrochloric acid erosion models, representing clinically relevant extrinsic and intrinsic sources of enamel erosion. These acids differ in their mechanisms of enamel dissolution, with citric acid exhibiting additional chelating properties. The purpose of including both models was not to directly compare their erosive potential, but instead to evaluate whether the protective effect of keratin was maintained under different erosive conditions. Keratin-treated samples demonstrated significantly greater hardness retention than the control groups under both models, suggesting that its protective effect is not limited to a specific type of acid challenge. As the intrinsic and extrinsic models differed in both acid chemistry and exposure protocol, the findings are best interpreted in terms of keratin's performance within each erosion model rather than as a comparison between the two acids.

Profilometric analysis demonstrated that keratin-treated samples exhibited the lowest mean surface loss under both erosion models, suggesting a trend towards greater protection against erosive challenge. However, the differences between treatment groups did not reach statistical significance and should therefore be interpreted with caution. In contrast to microhardness measurements, which reflect enamel softening and demineralisation, profilometry quantifies physical surface loss. Thus, while both techniques demonstrated trends consistent with a protective effect of keratin, the present findings provide stronger evidence for protection against enamel softening than for the prevention of measurable tissue loss.

**Figure 7:**
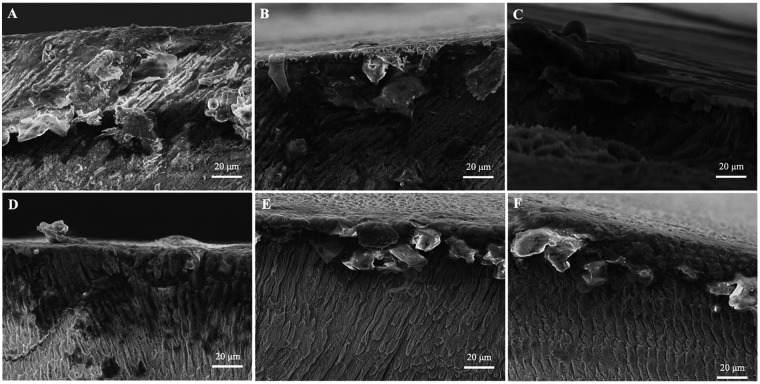
Scanning electron microscopy (SEM) images of treated enamel window following hydrochloric acid exposure. SEM images are shown for each treatment group: **(A)** deionised water, **(B)** artificial saliva, **(C)** 1,450 ppm fluoride, **(D)** 5,000 ppm fluoride, **(E)** 22,600 ppm fluoride and **(F)** keratin. Images were captured at ×750 magnification, with a working distance of 10 mm, acceleration voltage of 10.0 kV, and a probe current of 6.

SEM imaging provided qualitative support for these findings by indicating improved preservation of enamel morphology in the keratin- and high-fluoride treated groups compared with the controls. In the citric acid model, treated enamel surfaces appeared smoother and less porous than controls, and raised, globular deposits consistent with residual treatment layers were visible on both keratin- and fluoride-treated samples (Figure 4) (39). Cross-sectional SEM confirmed these observations, revealing preserved subsurface enamel architecture in the treated groups, whereas control samples displayed pronounced demineralisation and collapse of enamel rods (Figure 6) (38). Similar patterns were observed under hydrochloric acid exposure: keratin-treated surfaces appeared the smoothest and most cohesive, while controls exhibited irregular erosion and exposed enamel prisms (Figure 5). At a subsurface level, enamel architecture in hydrochloric acid exposed samples were similarly preserved (Figure 7). Together, these SEM findings suggest the presence of a surface layer associated with the keratin treatment that may have contributed to limiting acid-induced surface and subsurface demineralisation. The preservation of enamel morphology observed in the keratin-treated specimens was similar to that seen in the 22,600 ppm fluoride group, supporting further investigation of keratin as a potential biomaterial for erosion prevention. This interpretation is consistent with previous studies of mineralised keratin membranes, which demonstrated spherulitic growth and facilitated the nucleation and subsequent growth of hydroxyapatite crystals (28, 33). Furthermore, EDS and elemental mapping performed in these studies confirmed calcium and phosphate retention within the keratin-mediated layer. However, as compositional analyses were not performed in the present study, the chemical identity of the observed surface layer could not be directly confirmed.

The present study suggests that keratin is capable of forming a protective surface coating and enhancing resistance to acid induced enamel softening under controlled *in vitro* conditions. It is important that these observations are distinguished from the clinical translation of keratin-based therapies as despite these demonstrated effects, a number of factors remain to be established before clinical application can be considered. These include coating stability and retention within the oral environment, formulation development, delivery method, patient acceptability and long term safety. Consequently, the present results should be regarded as proof-of-concept observations that require further *in situ* and clinical validation before clinical translation can be considered.

In interpreting the results, several limitations should be acknowledged. As the enamel specimens were derived from extracted human teeth, natural biological variability between samples, including potential age-related differences in enamel properties, may have influenced baseline variability. However, baseline measurements were obtained for each specimen and treatment effects were assessed relative to these baseline values, thereby helping to minimize the influence of inter-specimen variability on the interpretation of treatment outcomes. The *in-vitro* model, although carefully controlled, cannot fully replicate intraoral factors such as salivary flow, pellicle regeneration, mechanical wear, or pH fluctuations. The preparation of enamel samples through polishing and embedding also presents a potential limitation. While this approach was necessary to standardise surface topography, it may have removed the aprismatic surface layer, which is naturally more mineralised and acid-resistant, potentially exaggerating erosion compared with intact enamel. Furthermore, SEM was solely used as a qualitative assessment technique, with one specimen from each treatment group randomly selected for surface imaging and one specimen selected for cross-sectional imaging. Although the SEM observations supported the microhardness and profilometry findings, examination of a greater number of specimens and incorporation of quantitative image analysis or blinded scoring would strengthen the morphological observations.

Future studies should employ larger sample sizes, repeated or cyclic acid exposures, and longer-term testing to evaluate treatment durability under conditions more representative of clinical erosion. Complementary techniques, such as coupling SEM with energy-dispersive x-ray spectroscopy, would provide valuable elemental data on calcium and phosphate retention, help characterise the composition of the observed surface layers and further clarify the underlying mechanisms of protection. In addition, exploring combined keratin-fluoride formulations could also determine whether synergistic effects occur and whether their combined use enhances enamel protection beyond either treatment alone.

Overall, keratin consistently conferred measurable protection against acid-induced enamel softening and demonstrated a trend towards reduced surface loss. Microhardness, profilometry, and SEM data collectively demonstrate that keratin forms a thin, protective surface layer that limits demineralisation, preserves surface morphology, and maintains subsurface integrity. These findings suggest that keratin may represent a potential biomaterial for erosion prevention and provide a foundation for future *in situ* and clinical investigations to evaluate its effectiveness under oral conditions.

## Data Availability

The raw data supporting the conclusions of this article will be made available by the authors, without undue reservation.
