# Supplementary material for: Keratin-based protection of enamel against acid-erosion
Source: Front Dent Med. 2026 Jul 13;7:1869803. doi: 10.3389/fdmed.2026.1869803 (PMC13402517; doi:10.3389/fdmed.2026.1869803)
Supplement: Supplementary file 1 [file Datasheet1.pdf]

Supplementary Material

1 Supplementary Tables

Group	Citric: Post-treat	Citric: Post-acid	HCl: Post-treat	HCl: Post-acid
**Keratin**	76.56 ± 8.86	86.96 ± 7.91	46.87 ± 19.10	69.94 ± 6.48
**1450 ppm NaF**	54.68 ± 12.74	65.07 ± 5.03	32.05 ± 9.35	57.75 ± 5.99
**5000 ppm NaF**	43.47 ± 13.45	68.60 ± 6.43	48.33 ± 9.21	61.92 ± 8.02
**22,600 ppm NaF**	51.98 ± 10.06	74.70 ± 4.12	37.29 ± 13.85	64.26 ± 9.11
**Artificial Saliva**	92.95 ± 2.02	52.74 ± 12.14	76.32 ± 7.99	52.76 ± 5.03
**Deionised Water**	88.40 ± 3.69	41.32 ± 8.27	75.17 ± 7.90	51.08 ± 5.40

Supplementary Table 1. Percentage enamel microhardness retained following treatment and acid exposure. Mean ± standard deviation of percentage enamel microhardness retained following treatment application and subsequent acid exposure. Values are presented for all experimental groups under citric acid and hydrochloric acid (HCl) erosion models. Post-treat denotes microhardness measured following treatment application, while post-acid denotes microhardness measured following acid exposure.

Group	Citric: Post-treat	Citric: Post-acid	HCl: Post-treat	HCl: Post-acid
**Keratin**	0.41 ± 0.32	-5.07 ± 2.82	0.84 ± 0.73	-1.72 ± 2.90
**1450 ppm NaF**	0.96 ± 3.91	-5.34 ± 1.58	2.75 ± 2.52	-3.92 ± 2.29
**5000 ppm NaF**	-7.57 ± 5.87	-5.60 ± 0.69	-4.14 ± 4.39	-2.39 ± 1.81
**22,600 ppm NaF**	-2.42 ± 2.70	-5.42 ± 1.50	-4.33 ± 3.35	-3.49 ± 1.81
**Artificial Saliva**	-0.18 ± 0.67	-6.66 ± 1.57	0.12 ± 0.08	-5.15 ± 2.41
**Deionised Water**	-1.11 ± 1.27	-6.95 ± 2.15	-0.20 ± 0.88	-3.90 ± 2.02

Supplementary Table 2. Enamel step height following treatment application and acid exposure. Mean ± standard deviation of enamel step height measured following treatment application and subsequent acid exposure for all experimental groups under citric acid and hydrochloric acid (HCl) erosion models. Post-treat denotes step height measured followed treatment application, while post-acid denotes step height measured following acid exposure. Step height values are expressed in µm.

Sample No.	Treatment	Acid Model	Baseline KHN	Post-Treatment KHN	Post-Acid KHN
**47**	Keratin	Citric acid	383.86	275.72	328.75
**55**	Keratin	Citric acid	399.08	333.16	384.87
**62**	Keratin	Citric acid	398.26	269.9	303.88
**65**	Keratin	Citric acid	358.53	316.82	332.70
**70**	Keratin	Citric acid	366.01	261.09	306.13
**1**	Keratin	HCl	389.74	122.89	308.35
**3**	Keratin	HCl	363.24	198.72	321.50
**4**	Keratin	HCl	393.5	277.82	325.37
**8**	Keratin	HCl	393.79	163.26	331.42
**72**	Keratin	HCl	385.38	319.64	391.07
**26**	1450 ppm NaF	Citric acid	380.67	200.14	273.28
**27**	1450 ppm NaF	Citric acid	395.89	138.07	262.88
**48**	1450 ppm NaF	Citric acid	385.26	223.24	254.26
**57**	1450 ppm NaF	Citric acid	385.69	224.47	243.83
**66**	1450 ppm NaF	Citric acid	371.30	259.26	215.25
**23**	1450 ppm NaF	HCl	385.52	121.20	246.32
**58**	1450 ppm NaF	HCl	397.61	128.11	263.00

**61**	1450 ppm NaF	HCl	399.90	224.41	294.11
**69**	1450 ppm NaF	HCl	372.01	139.64	262.62
**71**	1450 ppm NaF	HCl	372.93	131.48	269.67
**7**	5000 ppm NaF	Citric acid	390.47	146.22	259.26
**10**	5000 ppm NaF	Citric acid	394.66	157.59	277.19
**13**	5000 ppm NaF	Citric acid	385.83	143.88	239.80
**15**	5000 ppm NaF	Citric acid	321.13	216.30	253.30
**63**	5000 ppm NaF	Citric acid	399.12	141.09	260.70
**14**	5000 ppm NaF	HCl	387.34	241.13	269.46
**38**	5000 ppm NaF	HCl	389.79	233.74	282.14
**53**	5000 ppm NaF	HCl	285.81	119.38	240.83
**54**	5000 ppm NaF	HCl	378.31	246.96	296.17
**67**	5000 ppm NaF	HCl	358.17	217.55	240.06
**9**	22,600 ppm NaF	Citric acid	352.20	215.70	244.86
**34**	22,600 ppm NaF	Citric acid	382.72	195.66	280.97
**37**	22,600 ppm NaF	Citric acid	349.03	221.11	282.15
**42**	22,600 ppm NaF	Citric acid	308.87	113.21	234.10

**43**	22,600 ppm NaF	Citric acid	234.93	119.33	173.69
**17**	22,600 ppm NaF	HCl	339.30	152.35	293.69
**19**	22,600 ppm NaF	HCl	393.34	117.92	310.38
**30**	22,600 ppm NaF	HCl	381.30	264.55	312.96
**44**	22,600 ppm NaF	HCl	398.48	140.97	259.89
**64**	22,600 ppm NaF	HCl	358.25	162.00	267.48
**28**	Artificial saliva	Citric acid	399.54	378.43	228.07
**32**	Artificial saliva	Citric acid	387.91	366.61	194.70
**39**	Artificial saliva	Citric acid	296.85	278.84	210.21
**49**	Artificial saliva	Citric acid	382.51	345.30	146.57
**68**	Artificial saliva	Citric acid	366.10	334.29	173.05
**5**	Artificial saliva	HCl	392.73	341.42	247.50
**6**	Artificial saliva	HCl	389.61	370.97	251.18

**21**	Artificial saliva	HCl	311.27	285.04	201.63
**59**	Artificial saliva	HCl	387.78	369.82	226.64
**60**	Artificial saliva	HCl	398.26	340.08	262.14
**11**	Deionised water	Citric acid	388.71	319.27	133.09
**12**	Deionised water	Citric acid	399.52	352.62	138.46
**16**	Deionised water	Citric acid	399.94	358.06	217.63
**22**	Deionised water	Citric acid	399.48	364.79	173.96
**36**	Deionised water	Citric acid	389.76	353.75	154.84
**24**	Deionised water	HCl	324.65	281.72	212.55
**29**	Deionised water	HCl	382.20	315.61	220.46
**40**	Deionised water	HCl	366.16	334.66	215.43
**46**	Deionised water	HCl	362.34	339.56	237.16
**56**	Deionised water	HCl	395.52	381.83	233.60

Supplementary Table 3. Raw Knoop hardness values for individual enamel specimens at baseline, after treatment application and following acid exposure. Values represent the mean Knoop Hardness Number (KHN) calculated from five indentations per specimen.

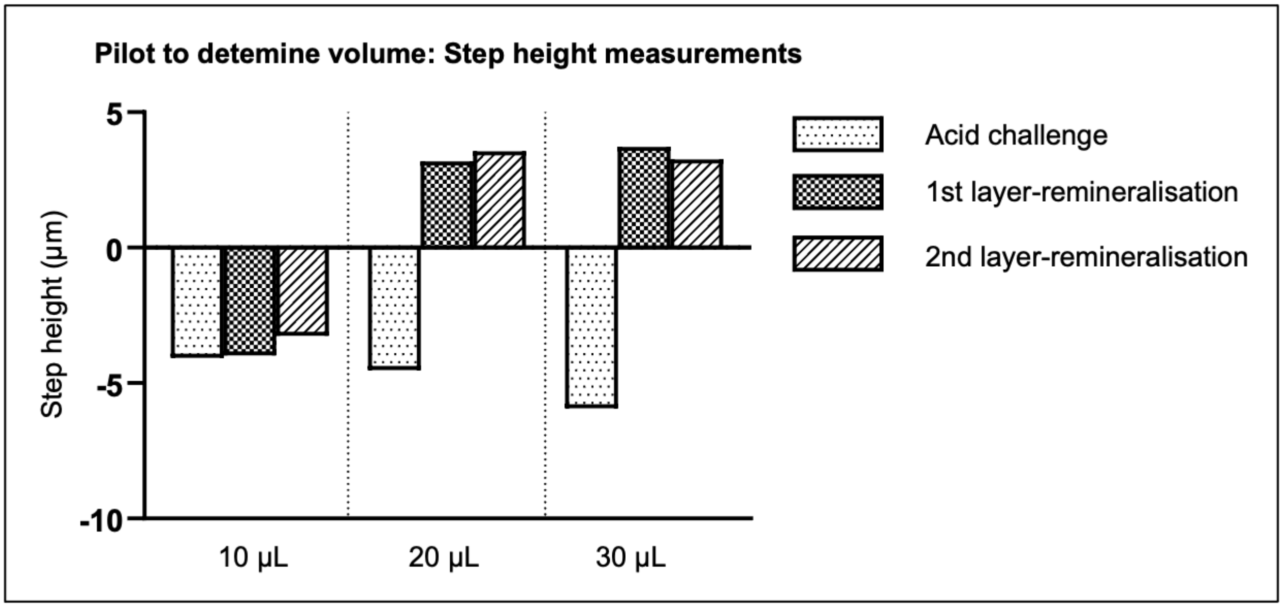

Supplementary Figure 1. Step height measurements from the preliminary optimisation study used to determine the keratin application volume. Measurements were recorded following acid challenge and after successive keratin applications using 10, 20 and 30 µl volumes.
